# The thymic flap for bronchial stump reinforcement following lobectomy

**DOI:** 10.1186/1753-6561-9-S1-A44

**Published:** 2015-01-14

**Authors:** MA Wilson, ME O’Donnell, SD Cassivi, C Seder

**Affiliations:** 1 Royal College of Surgeons in Ireland, 123 St. Stephen’s Green, Dublin 2. Ireland; 2 Division of Vascular and Endovascular Surgery, Mayo Clinic, Arizona, USA; 3 Division of Thoracic Surgery, Mayo Clinic, Rochester, USA

## Background

Reinforcement of the bronchial stump following upper lobectomy has been reported to decrease the prevalence of bronchopleural fistula [1]. A parietal pleural flap remains the mainstay of surgical support for such cases.

## Methods

We present the successful use of the right inferior pole of the thymus for bronchial coverage following upper lobectomy due to extensive pleural adhesions precluding conventional flap coverage.

## Results

A sixty-one year old male presented with a one-day history of severe left chest pain and a five-week history of a non-productive cough. He had a history of multiple bilateral rib fractures following a motor vehicle accident. He was an ex-smoker with no previous asbestos exposure. His respiratory rate was 22/minute with room air oxygen saturation of 95%.He had absent left basal breath sounds. Blood tests were normal. An erect chest x-ray revealed a left sided pleural effusion and a 2.6cm right upper lobe mass, confirmed with CT imaging..Although bronchoscopy and thoracocentesis were negative for malignancy, transbronchial endoscopic ultrasound needle aspiration of station 4R lymph nodes reported non-small cell lung carcinoma (T1B,M0,N2).He responded to neoadjunctive chemoradiotherapy. Follow-up PET/CT imaging showed a reduction in the apical mass to 2.2cm. Right upper lobectomy was performed via a 5th rib-space posterolateral thoracotomy where a solitary malignant intrapulmonary peribronchial lymph was identified. Due to extensive pleural adhesions from previous rib fractures, the right inferior tip of the thymus was mobilized from the pericardium and retrosternal attachments and used to secure the bronchial stump. The patient remains well following an uneventful recovery. Post-lobectomy bronchopleural fistula remains a rare and serious complication with an incidence rate between 0.5%-0.99% [2].Persistent empyemas necessitating open drainage and prolonged hospitalization contribute to a high mortality rate ranging from 25%- 67% [2]. A reduction in complications had been reported with the incorporation of pleural,diaphragmatic, intercostal and azygous vein bronchial stump reinforcements [1]. In our case, the thymic flap was mobilized due to inability to successfully dissect the parietal pleura. Infante et al (2004) evaluated the protection of right pneumonectomy bronchial sutures with a pedicled thymus flap where 82% (27/33) of cases had a satisfactory thymic inferior pole length [3].

## Conclusions

The thymic flap appears to be a valid conduit for reinforcement of the bronchial stump particularly in patients with extensive pleural adhesions limiting mobilization of the other traditional flaps.
